# High-Precision Polishing of Fused Silica Microfluidic Chips via CO_2_ Laser

**DOI:** 10.3390/mi17020173

**Published:** 2026-01-28

**Authors:** Yuhan Cui, Qiuchen Xie, Qian Yu, Gang Wang, Weijia Guo, Tianfeng Zhou

**Affiliations:** 1 School of Medical Technology, Beijing Institute of Technology, Beijing 100081, China; yuhancui@sina.com (Y.C.);; 2 State Key Laboratory of Chips and Systems for Advanced Light Field Display, Beijing Institute of Technology, Beijing 100081, China; 3 School of Mechanical Engineering, Beijing Institute of Technology, Beijing 100081, China; 4 School of Optics and Photonics, Beijing Institute of Technology, Beijing 100081, China

**Keywords:** CO_2_ laser polishing, microfluidic chip, fused silica, high-precision

## Abstract

To address the severe surface imperfections induced during ultrafast pulsed laser fabrication of fused silica microfluidic chips, a high-precision CO_2_ laser polishing strategy based on shallow-layer melting and reflow was employed. This method enables localized melting within an extremely thin surface layer, effectively smoothing the topography without altering the original microstructure geometry. An L9(3^3^) orthogonal experimental design was conducted to systematically investigate the influence of key parameters on polishing quality, identifying defocus distance as the dominant factor affecting surface roughness, followed by scanning speed and laser power. The optimal parameter combination was determined to be a laser power of 8 W, a defocus distance of 6 mm, and a scanning speed of 5 mm/s. Furthermore, an overlap rate between 38% and 63% was found to ensure sufficient fusion without excessive remelting, with the minimum surface roughness of 0.157 µm achieved at a 50% overlap rate. Based on the optimized parameters, adaptive scanning paths were designed for different functional units of a fused silica microfluidic chip. Surface characterization demonstrated that the surface roughness was remarkably reduced from 303 nm to 0.33 nm, meeting optical-grade surface quality requirements.

## 1. Introduction

Microfluidic chips integrate multiple operational units involved in chemical and biological processes, including sample preparation, reaction, separation, and detection, onto a single chip with an area of only a few square centimeters or less [1]. This is achieved through functional microstructures, such as microgrooves and microchannels, fabricated on the substrate surface [2]. Owing to their advantages of convenience, rapidity, and high accuracy, microfluidic chips hold significant potential for biomedical applications, particularly in medical diagnostics and analytical testing [3].

Typical materials for microfluidic chips include polymers, paper-based substrates, silicon, hydrogels, and glass. Among these, glass stands out as an ideal material for high-sensitivity optical detection and reusable microfluidic devices due to its chemical inertness, high optical transmittance, low fluorescence background, and excellent surface stability [4]. Within the category of glass materials, fused silica has become a critical substrate material for high-end microfluidic chips, characterized by its low metallic impurity content, strong resistance to acids and alkalis, extremely low coefficient of thermal expansion, and outstanding ultraviolet transmittance [5]. However, the high hardness and brittleness of fused silica make the fabrication of micro and nanostructures exceedingly difficult.

In the microfabrication of fused silica, wet etching employing hydrofluoric acid (HF) is widely utilized due to its rapid etching rate and suitability for batch processing. However, as an isotropic process, it is incapable of producing microstructures with high aspect ratios or vertically defined sidewalls [6,7]. To address this limitation, dry etching techniques have been adopted. Among these, reactive ion etching (RIE) achieves anisotropic etching by synergizing the physical sputtering of high-energy ions with the chemical reactions of reactive radicals, thereby enabling the fabrication of vertical sidewall profiles [8,9,10]. Nevertheless, its relatively low etching rate, high equipment cost, and complex process conditions limit its application in large-area microstructure fabrication. Electrochemical discharge machining (ECDM) removes material through localized, high-temperature ablation caused by spark discharges. But the exceptional chemical stability of fused silica results in significantly lower efficiency, with a material removal rate typically only one-third that of borosilicate glass [11]. Mechanical machining removes materials through mechanical forces using abrasives or cutting tools. While offering advantages in high flexibility and low cost, this method is constrained by low processing speeds and rapid tool wear [12,13].

In contrast, laser machining, particularly ultrafast (e.g., femtosecond or picosecond) pulsed laser machining, has emerged as a highly promising technique for fused silica microfabrication [14,15,16,17]. This non-contact method offers exceptional flexibility and high precision, achieving material removal via nonlinear absorption. However, a significant drawback is the formation of rough structures caused by melt expulsion and resolidification, which severely degrades optical performance and device functionality [18,19]. Consequently, high-precision polishing of the machined microstructures is indispensable.

Traditional mechanical polishing struggles to achieve high-precision polishing of three-dimensional structures in microscale and often introduces subsurface damage. Although wet etching polishing can improve surface roughness, the original microstructure morphology is inevitably altered. CO_2_ laser, with a wavelength of 10.6 μm, enables non-contact polishing and surface repair by inducing localized heating, material remelting, and reflow [20]. Since fused silica exhibits strong absorption characteristics in the 8–11 μm wavelength band, this study employs a CO_2_ laser for high-precision polishing of fused silica microfluidic chips fabricated by picosecond laser. The effects of key parameters such as laser power, defocus distance, scanning speed, and overlap rate on the polishing quality were systematically analyzed through orthogonal experiments; subsequently, the feasibility of this approach was validated via chip performance testing. This work aims to provide a novel technical pathway for the high-quality manufacturing of fused silica microfluidic chips.

## 2. Methods

### 2.1. Mechanism of CO_2_ Laser Polishing

**Figure 1:**
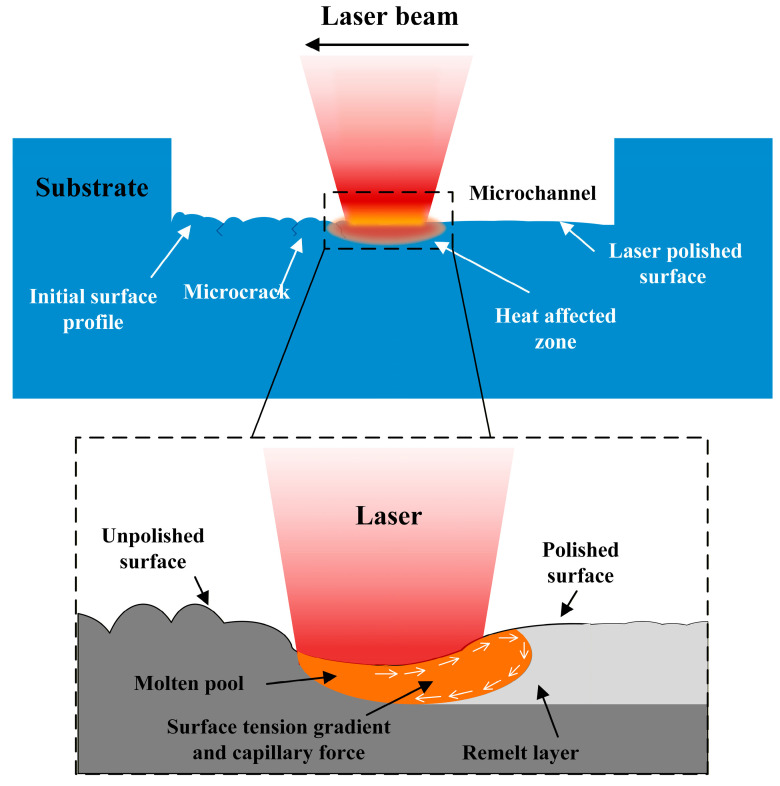
Schematic of the CO_2_ laser polishing principle for microfluidic chips.

The principle of CO_2_ laser polishing of fused silica is illustrated in Figure 1, which is essentially a surface reflow process based on a shallow-layer melting mechanism. When a high-energy-density CO_2_ laser beam is focused onto the wall of a microfluidic chip, the surface material absorbs the laser energy, causing its temperature to rise rapidly until it reaches a molten or vaporized state. As the laser beam moves, the molten material is redistributed under the action of surface tension, resulting in a smooth surface [21]. Simultaneously, the thermal effect of the CO_2_ laser can eliminate microcracks and defects induced by the thermal processes during ultrafast laser machining, thereby improving the overall surface quality.

It is worth noting that during CO_2_ laser polishing, the energy density of a continuous laser is much higher than that of a pulsed laser, which induces fluid convection within the molten pool on the polished surface. As the laser moves forward along the preset polishing direction, the molten pool gradually solidifies as the laser beam moves away.

Owing to the high absorption coefficient of fused silica at the wavelength of 10.6 μm, the material can rapidly absorb laser energy and convert it into heat within an extremely shallow depth when irradiated by a CO_2_ laser, leading to rapid surface heating and localized melting. The entire process can be described by the transient heat conduction equation.

(1) $$\rho C_{p}\frac{\partial T}{\partial t}=\nabla \cdot (k\nabla T)+Q(x,y,z,t)$$

where ρ is the material density, $C_{p}$ is the specific heat capacity, *k* is the thermal conductivity, *T* is the temperature, *t* is the time, and Q(x,y,z,t) is the volumetric laser heat source term.

The energy deposition caused by laser irradiation is highly concentrated at the material surface, thereby forming a micrometer-scale molten zone within the superficial layer. Its depth can be approximated by a thermal diffusion model:

(2) $$h\approx \sqrt{4\kappa t_{p}}$$

where $\kappa =k/(\rho C_{p})$ is the thermal diffusivity and $t_{p}$ is the effective laser interaction time. This expression reflects that the formation of the molten layer is a typical shallow-layer melting behavior. When the energy density F increases above the material’s melting threshold $F_{th}$, the melt depth increases logarithmically with the energy density. This indicates that the polishing process is highly sensitive to energy control, and a slight excess in energy can lead to the deepening of the molten pool, thereby inducing undesirable deep-melting effects, including severe remelting, thermal stress cracks, bubbles and pores.

(3) $$h\propto ln(\frac{F}{F_{th}})$$

Within an appropriate range of energy density, laser-induced melting occurs primarily at the microscopic asperities on the surface. At this stage, the flow of the molten material is predominantly driven by surface tension. Under the action of capillary forces, the material flows from convex regions with high curvature to concave valleys with low curvature, achieving spontaneous surface smoothing. This process can be described by a thin-film flow model:

(4) $$\eta \frac{\partial h}{\partial t}=\gamma \nabla^{2}h$$

where η is the viscosity of fused silica, γ is the surface tension, h(x,y) describes the surface height profile, and $\nabla^{2}h$ represents the surface curvature. This relationship indicates that the surface tension gradient is the primary driving force for material reflow during the polishing process.

However, when the laser power is excessively high or the scanning speed is too low, a pronounced temperature gradient develops within the molten pool, inducing Marangoni convection [22]. Since the surface tension of glass decreases with increasing temperature, the molten material flows rapidly from the high-temperature zone to the low-temperature zone. The driving force for this flow can be expressed as

(5) $${\vec{F}}_{Ma}=-\frac{d\gamma }{dT}\nabla T$$

where ∇T is the temperature gradient. This type of convection is typically stronger and operates on a larger scale than capillary-driven reflow. It generates recirculating flows within the molten pool, leading to material redistribution over a broader area and potentially creating defects such as periodic polishing striations, ripple structures, or surface inhomogeneities. This implies that CO_2_ laser polishing exhibits a distinct optimal energy window.

After the laser beam passes, the molten material solidifies rapidly at a cooling rate related to the thermal diffusivity and the melt layer thickness. Rapid solidification helps to ‘freeze’ the smoothed morphology after reflow, enabling the polished surface to retain high flatness and low roughness. Meanwhile, the cooling process can eliminate microcracks and heat-affected zones generated during ultrafast laser machining, thereby further improving the optical quality.

Therefore, CO_2_ laser polishing of fused silica is a synergistic process involving laser energy absorption, shallow-layer melting, capillary-driven reflow, and rapid resolidification. By carefully regulating the laser energy density, scanning speed, defocus distance, and overlap rate to confine the shallow-layer melting to an optimal range, high-quality polishing can be achieved without inducing secondary damage.

### 2.2. Experimental Procedure of CO_2_ Laser Polishing

**Figure 2:**
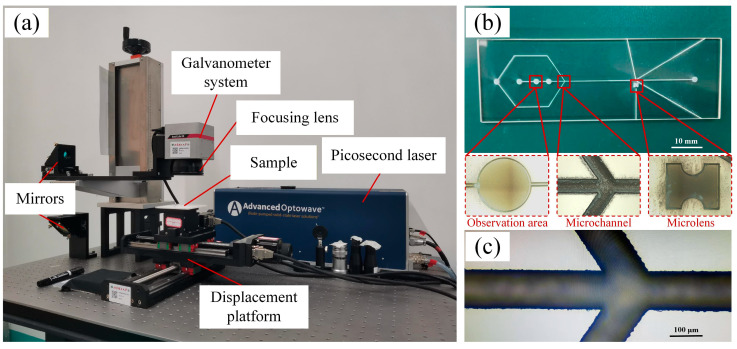
Fabrication of a fused silica microfluidic chip by picosecond laser direct-write etching: (**a**) fabrication setup, (**b**) fabricated sample, and (**c**) morphology observation.

This study employs a microfluidic chip designed for flow cytometry as the research subject, with key functional components including observation areas, an integrated microlens, and microchannels. As illustrated in Figure 2a, the substrate was initially patterned by direct-write etching using a 1064 nm picosecond laser (AMT-1064-20-100-W, Inno Laser, Shenzhen, China), operating at a focused spot diameter of 15 μm, a repetition rate of 100 kHz, a pulse width of 12 ps, and a single-pulse energy of 50 µJ. The fabricated chip is shown in Figure 2b. Optical microscopy of the etched microstructure edges revealed sharp profiles with minor edge chipping, attributed to stress concentration at these sharp features. These defects are expected to be eliminated via material remelting during the CO_2_ laser polishing.

**Figure 3:**
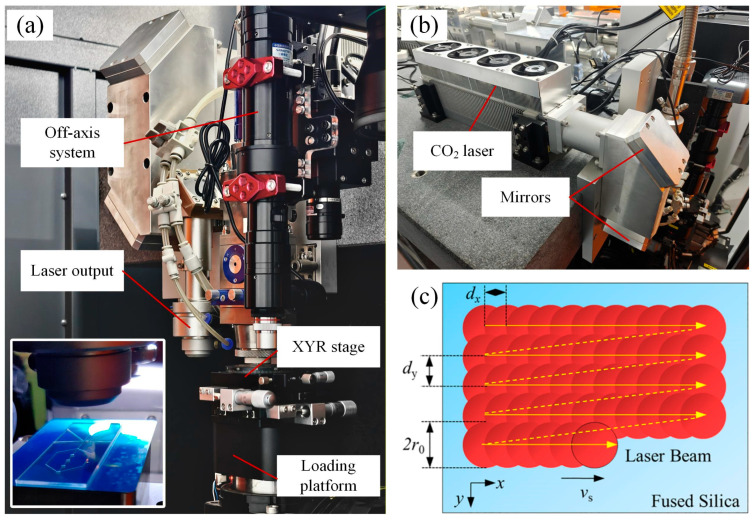
Polishing of a fused silica microfluidic chip using a CO_2_ laser: (**a**) polishing setup, (**b**) CO_2_ laser source, and (**c**) scanning path and overlap rate.

As shown in Figure 3, the polishing experiments were conducted using a CO_2_ laser (Diamond C-30A/GEM-30A, Coherent, Santa Clara, CA, USA). The polishing effect is primarily governed by several key factors such as laser power, duty cycle, defocus distance, scanning speed, and overlap rate.

The laser power and duty cycle directly determine the energy density at the focal point. Insufficient energy density fails to fully melt the material, thereby limiting the improvement in surface roughness, while excessive energy density can lead to surface over-remelting and even micro-bubble formation. Moreover, the CO_2_ laser beam features a typical Gaussian intensity distribution with a small focal spot; directly focusing it on the polishing surface would restrict the processed area and raise the risk of thermal damage. When the laser power is excessively low, reducing the defocus amount is necessary to concentrate the focal point as closely as possible on the fused silica surface to reach the material melting energy threshold, but this results in a limited polishing area per laser spot, which is unfavorable for high-efficiency, large-area polishing. In contrast, relatively high laser power enables the formation of a large polishing area via defocus amount adjustment, yet it cannot polish the microstructures on the microfluidic channels. Therefore, a specific defocus distance was intentionally set to shift the focal point away from the workpiece surface.

An L9 (3^3^) orthogonal experimental array was designed by selecting laser power, defocus distance, and scanning speed as the primary variables, with specific parameters listed in Table 1. Each parameter set corresponds to a distinct laser energy density distribution, and polishing was performed via single-line scanning. The optimal parameter combination was identified by analyzing the average surface roughness (Ra) and the range (*R*) value for each factor level.

In addition, this study analyzed the overlap rate to further investigate the heat accumulation and connection between adjacent polishing tracks. The overlap rate, which directly affects the energy accumulation between polishing lines and the resulting surface flatness, is determined by the laser frequency, scanning speed, and path spacing. During the scanning process, the laser focus forms a polishing spot with a radius of *r*_0_ on the workpiece surface. The step distance along the x-direction is *d*_x_, and the track offset along the y-direction is *d*_y_, as illustrated in Figure 3c. In the subsequent experiments, to ensure uniformity of the polished surface in both scanning directions, the overlap rates in the x- and y-directions were set equal. A parallel raster scanning pattern was employed, ensuring identical cooling time for each irradiated spot. By varying the overlap rate from 0% to 88%, the surface roughness and topographic profiles under different scanning conditions were obtained.

## 3. Results and Discussion

### 3.1. Effect of Laser Energy Density on Surface Polishing Quality

Theoretically, the laser power and the interaction time determine the energy deposited at the defect sites on the fused silica surface, which directly governs the melting and polishing outcome. The laser power is set by adjusting the duty cycle, while the interaction time is controlled by the moving speed of the stage. An increase in the duty cycle enlarges both the affected area and the melt depth after polishing. In contrast, a higher scanning speed reduces both the affected area and the melt depth. Consequently, precise control of the laser output power and interaction time, along with the selection of an appropriate defocus distance, is essential during the melt-polishing process to ensure effective polishing while minimizing adverse thermal effects on the component.

**Figure 4:**
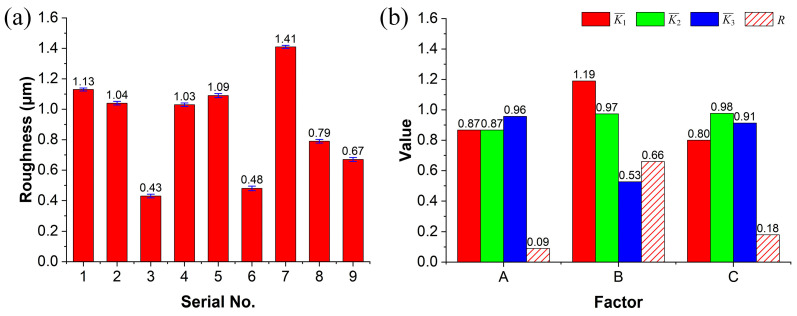
Analysis of laser energy density impact: (**a**) surface roughness under different parameter sets and (**b**) corresponding range analysis.

The surface roughness of single polishing lines corresponding to the parameter sets in Table 1 was measured three times and averaged, with the results presented in Figure 4a. Error bars in Figure 4a represent the standard deviation of the three repeated measurements. Both parameter sets 3 and 6 yielded superior surface quality, indicating that a moderate laser energy density facilitates the formation of a continuous and smooth molten layer, whereas insufficient or excessive energy density degrades the final polishing quality.

To further clarify the degree of influence of each factor and identify the optimal parameter combination, a range analysis was performed on the roughness data, as presented in Figure 4b. The results show that the defocus distance has the largest *R* value, signifying its most significant impact on polishing quality, followed by the scanning speed and finally the laser power. The defocus distance directly controls the spot size and energy density distribution, serving as the primary means of regulating melt depth and area. An excessively small defocus distance leads to overly concentrated energy, which can cause non-uniform remelting in micro-areas. In contrast, an appropriately increased defocus distance enlarges the spot area, resulting in a more uniform molten layer and facilitating the formation of a continuous, smooth flow interface. Consequently, the optimal parameter combination was determined to be Set No. 6, corresponding to a laser power of 8 W, a defocus distance of 6 mm, and a scanning speed of 5 mm/s.

### 3.2. Effect of Laser Overlap Rate on Surface Polishing Quality

**Figure 5:**
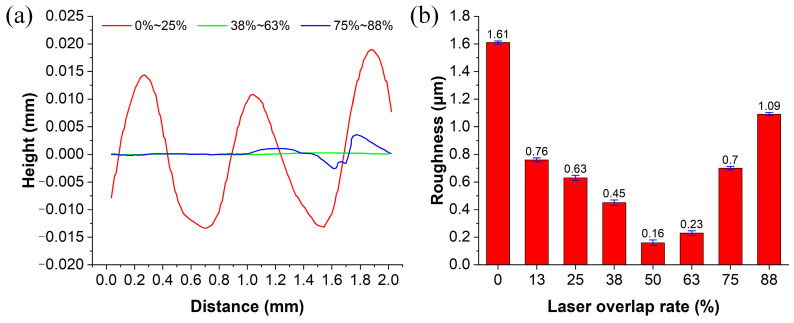
Analysis of laser overlap rate impact: (**a**) surface morphology and (**b**) average surface roughness under different laser overlap rates.

When polishing the structure surface of a microfluidic chip, the influence of the overlap rate between adjacent polishing paths on the final quality must also be considered. Figure 5a illustrates the evolution of surface morphology under different CO_2_ laser overlap rates. With an overlap rate in the range of 0~25%, the polished surface exhibits pronounced periodic height fluctuations. This indicates a lack of effective remelting and fusion between adjacent scan tracks, leaving distinct inter-track boundaries. As the overlap rate increases to 38~63%, the surface profile fluctuations diminish significantly, and the overall surface becomes notably flatter. This suggests sufficient fusion of the molten zones and moderate heat accumulation within this range, representing the optimal window for achieving high-quality polishing. When the overlap rate is further increased to 75~88%, although the overall surface remains relatively smooth, localized height disturbances emerge. Because an excessively high overlap rate causes the newly irradiated zone to remelt the previously smoothed layer, leading to a degradation in surface quality.

Figure 5b presents a quantitative analysis of the surface roughness at different overlap rates. The surface roughness values shown are the mean of three independent measurements; the error bars depict the standard deviation of these measurements. The results reveal that the surface roughness initially decreases and then increases with increasing overlap rate. The maximum roughness of 1.608 µm was observed at 0% overlap. As the overlap rate increased, the roughness decreased significantly, reaching a minimum of 0.157 µm at a 50% overlap. This indicates sufficient overlap and fusion between the molten zones of adjacent scan tracks, resulting in optimal surface flatness. However, when the overlap rate exceeded approximately 63%, the roughness exhibited a notable rebound. For instance, at an 88% overlap, the roughness increased to 1.085 μm, further verifying that excessive energy re-superposition deteriorates the polished surface. Therefore, a moderate overlap rate is crucial for obtaining a continuous and uniform polished surface, with the range of approximately 38~63% demonstrating the best overall performance. For the subsequent overall polishing of irregular structural units, the optimal overlap rate determined from these experimental results was applied to ensure uniform, continuous, and stable surface quality across the polished area.

### 3.3. Polishing Outcome of the Fused Silica Microfluidic Chip

The functional structures of the fused silica microfluidic chip include observation areas, an integrated microlens, microchannels, and optical fiber channels. Their geometric characteristics differ significantly, necessitating targeted path planning and energy control strategies during CO_2_ laser polishing. For long, groove-like structures such as microchannels and fiber channels, whose width is comparable to the CO_2_ laser polishing spot, a parallel raster scanning path can be employed. This allows for uniform energy deposition at the structure’s bottom, effectively removing roughness and repairing edge defects. The planning of the polishing path adopts an inward profile-filling strategy according to the microstructure contours by utilizing the path planning/path filling function of the equipment.

**Figure 6:**
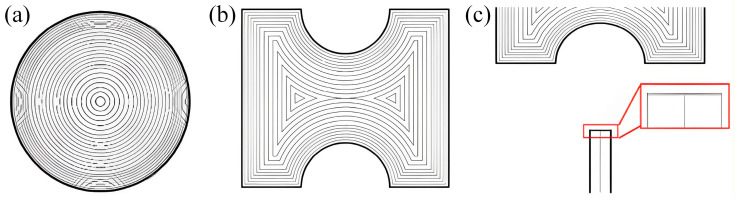
Adaptive path planning for CO_2_ laser polishing: (**a**) observation area, (**b**) microlens, and (**c**) optical fiber channel.

However, for the observation areas and the integrated microlens, which demand exceptional curvature continuity, using the same parallel scanning method would compromise their optical surface form and introduce curvature distortion. Thus, contour-following paths were designed to distribute the laser’s high-temperature zone along the curved surface, enabling continuous melt reflow and preserving the original optical curvature of the observation areas and microlens after polishing. As shown in Figure 6a,b, the path pattern features a higher density in the peripheral region and a lower density in the center. The longer peripheral paths cool earlier, making them susceptible to temperature gradients and residual stress, thus a higher path density is applied to induce a tempering-like reheating effect to mitigate potential cracks. Conversely, excessively dense paths in the central region would cause heat accumulation and bubble formation. Hence, the path spacing is gradually increased toward the center to promote stepwise cooling of the molten zone, resulting in a uniform and stable optical surface. Similarly, the end face of the optical fiber channel shown in Figure 6c requires multiple adjacent passes to achieve a smooth and molten surface.

**Figure 7:**
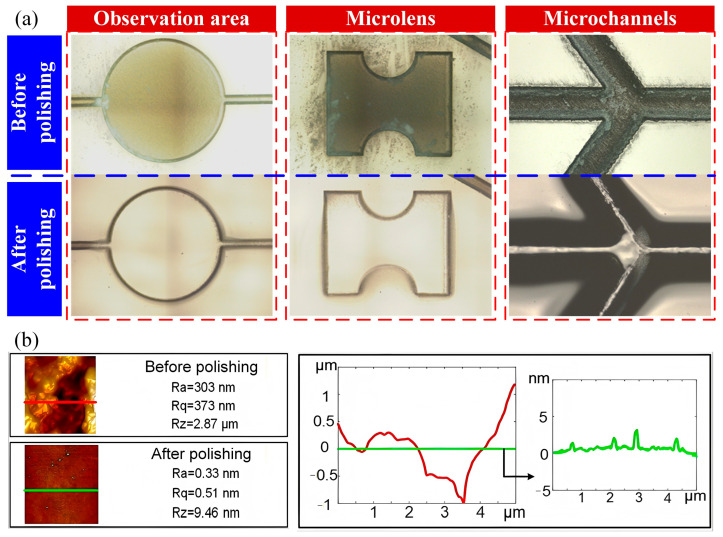
Comparison of the key functional microstructures on a fused silica microfluidic chip before and after CO_2_ laser polishing: (**a**) surface polishing results and (**b**) morphology characterization and roughness quantification.

Using the optimized CO_2_ laser polishing parameters and adaptive scanning paths described above, a comparison before and after polishing is shown in Figure 7a. The matte texture at the bottom of the observation area was effectively removed after polishing, exhibiting excellent transparency comparable to the pristine glass substrate. For the microlens structure, the base achieved high flatness, and its curved surface was polished, transforming the originally sharp edges into smooth transitions. This ensures the lens’s focusing capability during operation. The microchannels obtained a smooth bottom after polishing, which is crucial for reducing fluid flow resistance. The sharp edges created by the initial picosecond laser machining, which were prone to edge chipping, posed a risk of contaminating the fluid with tiny debris during chip operation. After polishing, these edges were rounded, and the chipping defects were eliminated through remelting.

The surface of the microstructures before and after CO_2_ laser polishing was further examined and quantified using laser confocal microscopy (LCM, VK-X100, Keyence, Osaka, Japan) and atomic force microscopy (AFM, DimensionIcon, Bruker, Billerica, MA, USA). As shown in Figure 7b, the surface roughness Ra was 303 nm before treatment but was dramatically reduced to 0.33 nm after polishing. Furthermore, the roughness profiles of the surface topography were plotted, confirming that the polished surface became more uniform and flat. These results demonstrate that the introduced CO_2_ laser polishing technique significantly improved the surface roughness of the on-chip glass microstructures, and the achieved Ra meets the international fabrication standard for optical components.

## 4. Conclusions

This study employed a high-precision CO_2_ laser polishing technique to achieve rapid, stable, and controllable polishing of fused silica microfluidic chips fabricated by ultrafast laser. A complete methodological framework encompassing parameter optimization, morphology regulation, and microstructure integrity was established, thereby providing an efficient and feasible technical pathway for the optical-grade fabrication of fused silica microfluidic chips. The main conclusions are as follows:

1. Defocus distance is the dominant factor governing polishing quality, primarily influencing melt depth, energy distribution, and surface reflow behavior, followed by scanning speed and laser power plays a comparatively minor role. An optimal parameter set of 8 W laser power, 6 mm defocus distance, and 5 mm/s scanning speed was identified.
2. Overlap rate critically affects energy uniformity and surface morphology continuity. A moderate overlap rate of 38~63% promotes continuous and uniform surface transitions, with the minimum surface roughness of 0.157 µm achieved at a 50% overlap. Insufficient overlap results in unfused regions, whereas excessive overlap leads to repeated remelting and surface disturbances.
3. Through localized shallow-layer melting induced by CO_2_ laser, rapid surface reflow driven by surface tension achieves significant smoothing without altering the microstructure geometry. The surface roughness was dramatically reduced from 303 nm to 0.33 nm, meeting the requirements for optical component fabrication.

While the above optimal parameters are subject to certain limitations, as they were obtained based on our specific experimental setup and sample morphology, the core value of this study lies in providing a viable solution to high-quality fabrication of fused silica microfluidic chips. For practical engineering implementation, further considerations must include the effects of temperature fluctuations, along with overall processing time and laser system costs.

## Figures and Tables

**Table 1:** Orthogonal experimental array of CO_2_ laser polishing.

Set No.	Factor A Laser Power (W)	Factor B Defocus Distance (mm)	Factor C Scanning Speed (mm/s)
1	6	4	5
2	6	5	9
3	6	6	7
4	8	4	9
5	8	5	7
6	8	6	5
7	10	4	7
8	10	5	5
9	10	6	9

## Data Availability

The data presented in this study are available on request from the corresponding author. The data are not publicly available as they are part of an ongoing long-term project.
